# Rank and Wormhole Attack Detection Model for RPL-Based Internet of Things Using Machine Learning

**DOI:** 10.3390/s22186765

**Published:** 2022-09-07

**Authors:** F. Zahra, NZ Jhanjhi, Sarfraz Nawaz Brohi, Navid Ali Khan, Mehedi Masud, Mohammed A. AlZain

**Affiliations:** 1 School of Computer Science (SCS), Taylor’s University, Subang Jaya 47500, Malaysia; 2 Computer Science and Creative Technologies, University of the West of England, Bristol BS16 1QY, UK; 3 Department of Computer Science, College of Computers and Information Technology, Taif University, Taif 21944, Saudi Arabia; 4 Department of Information Technology, College of Computers and Information Technology, Taif University, Taif 21944, Saudi Arabia

**Keywords:** RPL routing protocol, internet of things, RPL attacks, protocol-specific attacks, SN-inherited attacks, attack detection, machine learning

## Abstract

The proliferation of the internet of things (IoT) technology has led to numerous challenges in various life domains, such as healthcare, smart systems, and mission-critical applications. The most critical issue is the security of IoT nodes, networks, and infrastructures. IoT uses the routing protocol for low-power and lossy networks (RPL) for data communication among the devices. RPL comprises a lightweight core and thus does not support high computation and resource-consuming methods for security implementation. Therefore, both IoT and RPL are vulnerable to security attacks, which are broadly categorized into RPL-specific and sensor-network-inherited attacks. Among the most concerning protocol-specific attacks are rank attacks and wormhole attacks in sensor-network-inherited attack types. They target the RPL resources and components including control messages, repair mechanisms, routing topologies, and sensor network resources by consuming. This leads to the collapse of IoT infrastructure. In this paper, a lightweight multiclass classification-based RPL-specific and sensor-network-inherited attack detection model called MC-MLGBM is proposed. A novel dataset was generated through the construction of various network models to address the unavailability of the required dataset, optimal feature selection to improve model performance, and a light gradient boosting machine-based algorithm optimized for a multiclass classification-based attack detection. The results of extensive experiments are demonstrated through several metrics including confusion matrix, accuracy, precision, and recall. For further performance evaluation and to remove any bias, the multiclass-specific metrics were also used to evaluate the model, including cross-entropy, Cohn’s kappa, and Matthews correlation coefficient, and then compared with benchmark research.

## 1. Introduction

Due to the expansion of the internet of things (IoT) implementation in most disciplines at an alarming rate, it has been considered as a paramount technological revolution of this era. IoT is allegedly on the path to empowering the modern world by improving the efficiency and effectiveness of the systems in terms of saving time and automation of functions, while also introducing smartness in things. This state-of-the-art technology has markedly affected our lives by producing the notions of smart homes [1], smart cities [2], smart healthcare [3], and even wearable devices [4]. Furthermore, it has resulted in a significant impact on saving resources in urban and industrial domains [5,6].

The interaction and communication of intelligence-induced objects and people with each other anywhere at any given time has led to interconnected networks of billions of nodes in the world. This innovative technology is now embarking on machine-to-machine communication through the internet that will not necessarily require human intervention [7,8]. However, this would lead to various challenges at various levels in the complete IoT-enabled infrastructure. Primarily, the huge amounts of sensitive data communicated over the internet would fall victim to security attacks from various dimensions leading to compromised IoT-enabled infrastructures. Therefore, cybersecurity is one of the major challenges in IoT networks and systems.

The majority of the IoT devices are developed without considering security as a fundamental factor due to several reasons. Some of them include the small size of the device that cannot support complex mechanisms and resource constraints that do not allow computational overheads. Therefore, to address these gaps, the researchers are actively participating in proposing alternative solutions to improve the security of IoT-enabled smart systems. In particular, the network layer of these systems is studied and researched due to its high vulnerability to network and routing attacks.

The routing protocol for low-power and lossy networks (RPL) is a de facto protocol used in IoT networks. The Internet Engineering Task Force (IETF) [9] developed this protocol to address the unavailability of a routing standard that can fulfill the routing requirements and be implemented in resource-constrained, low-power, and lossy IoT networks. It works using the distance vector routing principle and forms a directed acyclic graph (DAG) or destination-oriented DAG (DODAG) to perform the routing functions. Five main control messages are responsible for developing a communication array. They are DODAG information solicitation (DIS), DODAG information object (DIO), destination advertisement object (DAO), DAO acknowledgement (DAO-Ack), and consistency check (CC) messages [10]. It also has local and global repair mechanisms responsible for repairing any inconsistencies, link outages, and other protocol-related problems. However, the lightweight core of the protocol and optional as well as specification-dependent security mechanisms lead to an increased potential for security attacks. These attacks are categorized into RPL-specific or protocol-specific attacks and sensor network (SN)-inherited attacks.

Rank attacks (RA) from the protocol-specific category and wormhole attacks (WHA) from the SN-inherited attack category are among the most threatening attacks targeting the security triad of the RPL network, which encompasses the confidentiality, availability, and integrity of the network and system. In a decreased rank attack, an attacker illegitimately broadcasts a smaller rank value to attract child nodes and becomes a preferred parent to launch the attack, while in a wormhole attack, two nodes form a tunnel and attract victim nodes to send their traffic to the sink node through their path. They then replay or selectively drop the packets, which in turn results in deterioration of the network topology, life, as well as resources. Therefore, it is crucial to address these attacks to improve the security in RPL-based IoT networks.

Machine-learning (ML) is an emerging paradigm for data analytics, automation, pattern recognition, anomaly detection, and prediction-related tasks. The techniques used in this discipline include statistical algorithms and models which have exhibited their effectiveness in different fields including cybersecurity. Moreover, ML is compatible with IoT systems in working principles and concerning data requirements because they generate a large amount of data that ML can utilize to build robust models. Therefore, this study proposes an ML-based model for addressing RA and WHA attacks in RPL-based IoT networks. The proposed model named MC-MLGBM leverages the light gradient boosting machine model to perform multiclass classification for attack detection where the main task is to classify the network traffic data into begin, RA, and WHA target classes. MC-MLGBM stands for multiclass machine-learning-based model leveraging the light gradient boosting machines for attack detection (MC-MLGBM).

Moreover, a novel dataset is generated as part of this study due to the scarcity of publicly available datasets in the domain of RPL-based IoT. It includes benign, RA, and WHA traces where the attack data were collected through extensive network modeling and simulation utilizing various network models. The network models are exclusively designed and implemented for the generation of the required network traffic dataset. Furthermore, the mobility factor is addressed in this paper using the random waypoint mobility model to create a mobile RPL-based IoT network environment. The model performance is evaluated using standard evaluation parameters and metrics including accuracy, precision, and recall. To further validate the results for multiclass classification, three other multiclass-specific metrics are used. They are cross-entropy, Cohn’s kappa, and Matthews Correlation Coefficient (MCC). Furthermore, the proposed model is compared with benchmark research and relevant classifiers. The experimental results present that the proposed model outperforms the benchmarks in terms of attack categorization, the number of attacks detected, mobility, and performance evaluation using ML parameters.

The main contributions of the paper are summarized as follows:

- A novel ML-based model is proposed for RPL-specific RA attack and SN-inherited WHA attack detection which is trained on a self-generated dataset. The parameters are optimized and characterized by high accuracy, a high detection rate, and high performance, which is assessed through standard ML evaluation metrics as well as multiclass classification evaluation metrics.
- A novel dataset is generated that consists of both RPL-specific and SN-inherited attacks in the static and mobile state of IoT nodes. The dataset is produced to address the lack of recent datasets in the RPL-based IoT domain.
- The light gradient boosting machine model is leveraged to perform multiclass classification for attack detection in RPL-based IoT.
- An in-depth evaluation of the MC-MLGBM model is carried out based on different evaluation metrics in the training (for learning purposes) and testing phases.

The remaining paper is organized as follows: Section 2 reviews the relevant existing literature, Section 3 presents the methodology and proposed model, Section 4 recounts the experimental results, and Section 5 discusses the conclusion, the limitations of this work, and future research direction.

## 2. Related Work

IoT networks are vulnerable to different security attacks and the RPL protocol impacts this vulnerability, which causes the RPL-based IoT network to become prone to RPL-specific or protocol-specific and SN-inherited attacks. The research in this domain is still in its initial stages, notwithstanding the several studies conducted regarding attack detection and prevention in such networks. RPL-based IoT networks are vulnerable to both protocol-specific and SN-inherited attacks simultaneously, which consumes the RPL-based network resources and threatens confidentiality, integrity, and availability (CIA) security triad requirements [11]. In this section the related existing literature is reviewed, which is presented by researchers for attack detection in RPL and IoT.

In [12], the authors have discussed recent communication and network protocols applicable in the IoT environment, while in [10], the authors have provided an in-depth analysis of RPL-related security attacks, RPL composition, components, and control messages. They have also presented the attack classification taxonomy and a structured categorization of countermeasures presented by other researchers. In [13], the authors have proposed a machine-learning-based binary classification method to detect one of the protocol-specific attack types. They have generated their dataset due to the lack of an appropriate dataset by creating a version number attack network model to simulate the attack in the Cooja network simulator and gather the data. In machine learning, feature scaling and selection are two of the most important steps and there are numerous techniques to perform these steps mentioned in the literature. The authors have used a min-max scaling procedure and forward feature selection technique to preprocess their dataset. Furthermore, a light gradient boosting machine is used as a binary classifier to detect the version attack in an RPL network with a different number of nodes. The results demonstrate that the proposed model performs exceptionally well in the classification of normal traffic from attack traffic. However, there is a gap identified in terms of addressing SN-inherited attacks and consideration of node mobility. The mobility metric is mentioned but not discussed.

Similarly, in [14], the authors have proposed a deep learning-based model for the detection of hello flood attacks. These attacks fall under the category of SN-inherited attacks. They have evaluated the model using accuracy and regression-related evaluation metrics including mean squared error, mean absolute error, and root mean squared error. The model is compared with other classifiers including support vector machines (SVM) and it performs well in comparison. However, the protocol-specific attacks are not considered in this research and the mobility of IoT nodes is also not addressed. Moreover, deep learning methods are well known for requiring more time and data, and their difficulty in interpretation. In [15], the authors have proposed to use the self-organizing map-based deep learning strategy for developing an intrusion detection system to address RPL attacks. However, the placement strategy—which is an important factor in such solutions—was not stated clearly in this study [13].

In [16], the authors proposed to address RPL attacks including the rank attack using a deep neural network approach. They generated the dataset using the Cooja simulator in the Contiki operating system and evaluated the model against standard performance parameters including accuracy, achieving the highest accuracy for one attack called the hello flood attack. However, this approach leads to similar problems, as discussed earlier in this section, i.e., long training time, and vulnerability to other attacks in the model layers.

In [17], the authors have proposed a trust-based mechanism to address security issues in RPL networks with a focus on protocol-specific rank attacks. They performed a simulation study to evaluate the proposed expected transmission count metric-based strategy in terms of energy consumption, packet delivery rate, throughput, and rank change in the network. However, mobility was not addressed. Additionally, a hardware security chip is required along with the nodes. In [18], the authors have addressed transmission attacks in vehicular ad hoc IoT networks using a trust-based technique. However, the routing attacks were not considered and it was limited to vehicular ad hoc networks. Similarly, in [19], the authors have addressed security issues in vehicular ad hoc IoT networks using a trust-based protocol for jamming attacks and identifying malicious nodes in such IoT-enabled networks. However, RPL attacks were not considered in the proposed approach.

Numerous review studies and surveys have been conducted for exploring different techniques to address the security attacks in IoT and RPL-based IoT. For instance, in [20], the authors have performed a systematic literature review of ML and DL strategies for attack detection in RPL-based IoT. Similarly, in [21], the authors have conducted a detailed survey for the evaluation of RPL attacks. They have also assessed various detection and mitigation methods using RPL control messages. Table 1 presents a summary of the related works in addition to the dataset(s) used, methodology, attacks considered, and limitations or research gaps.

## 3. Methodology

**Figure 1:**
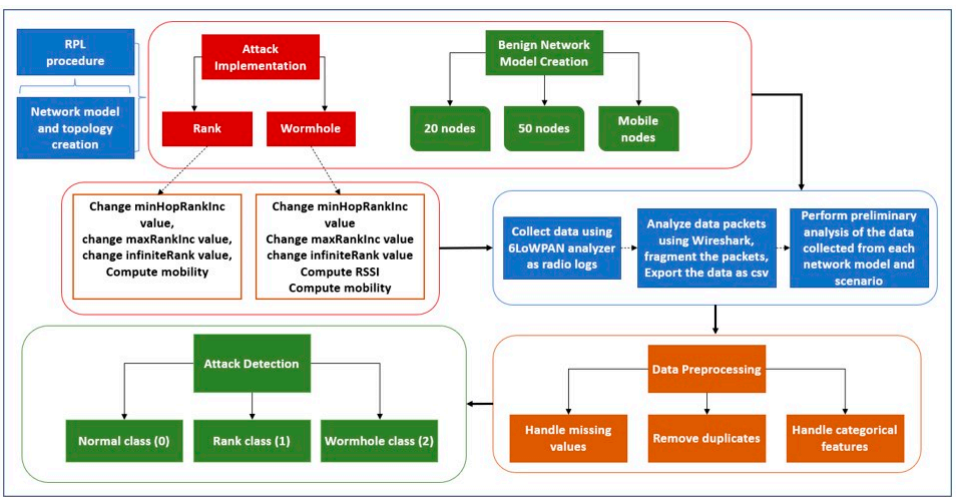
Conceptual design and architecture of the proposed model.

This paper proposes an attack detection model for the detection and classification of protocol-specific as well as SN-inherited attacks in RPL-based IoT networks using machine learning. The model is based on the following phases: network model simulation for data collection from different required scenarios, dataset creation and analysis, preprocessing and feature engineering, model development, and training. The trained model is assessed using the test dataset with a selection of appropriate performance metrics which are discussed in the forthcoming sections. Figure 1 presents the conceptual architecture and model design. The various modules in the previously discussed phases form a liaison to achieve the results in terms of attack detection and classification of attack type, which was then evaluated using several pertinent, multiclass-related metrics.

### 3.1. Network Model Setup, Simulation, and Network Scenarios for Data Collection

To collect the required data, the RPL-based IoT network is simulated depending on the type and amount of data needed. In this research study, we need benign network traffic data, protocol-specific attack data, and SN-inherited attack data. To experiment with generating and acquiring these types of data, we have used the Cooja network simulator which runs on the Contiki 3.0 operating system. This simulator supports RPL-based IoT network simulation inherently and allows the emulation of actual sensor node hardware with a variety of node types represented as motes during the simulation. The simulator is Java-based with foundations in C language [25]. For our network model simulation, we have used the latest stable release, Contiki 3.0 on an Oracle virtual machine called VirtualBox [26] with 8 GB of RAM and 60 GB of hard disk for raw data collection from different network models and scenarios. Each node emulates a sky mote and the benign network was simulated as soon as initiated. The two attack scenarios were simulated after the network achieves a certain level of stability. This step was performed to observe the maximum effect of the attack on the network and its resources. Table 2 presents the data generated from different network models and simulation scenarios. In the next sub-section, the various network models are discussed which were implemented for gathering the desired data.

### 3.2. Simulation of Benign Network Models, Protocol-Specific Attack Models, and SN-Inherited Attack Models

**Figure 2:**
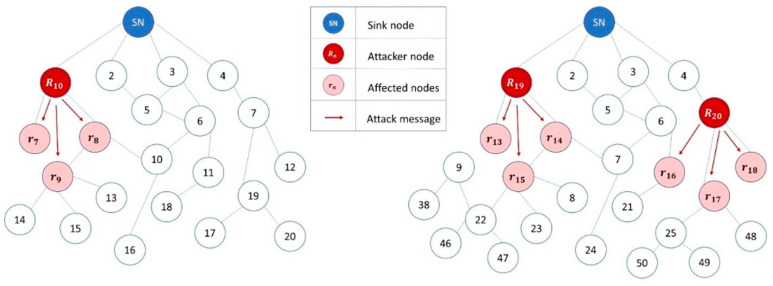
Protocol-specific attack simulation in RPL-based IoT networks simulated in Cooja. The attacker is located at position 10 in the left-hand side network, while two attackers are located at positions 19 and 20 in the right-hand side network.

**Figure 3:**
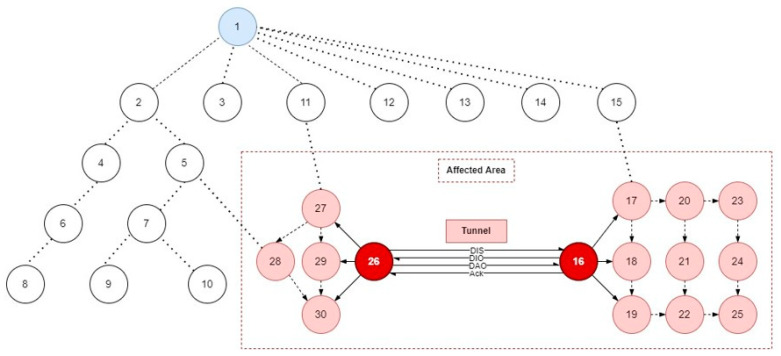
SN-inherited attack simulation in RPL-based IoT network simulated in Cooja.

In this paper, we have proposed to address protocol-specific and SN-inherited attack types. Therefore, three network models are developed inclusive of the benign model to collect the normal network traffic as a benchmark followed by attack traffic generation and collection. The algorithms for protocol-specific (RA) attacks and SN-inherited (WHA) attacks are presented in Algorithm 1 and Algorithm 2, respectively. The attack scenarios are illustrated in Figure 2 and Figure 3.

#### 3.2.1. Benign Network Model Simulation

The benign network model is simulated for benchmarking purposes, and two scenarios are considered for data collection. In the first scenario, the number of nodes is increased from twenty to fifty, while in the second scenario, the state of the node is considered, which is either static or mobile. The data were collected from two use cases of each scenario and used as a baseline dataset against attack datasets. The simulations were implemented in random and grid positionings backed by the methods used by researchers in the existing relevant literature which can be referred to in [13,27,28,29]. The raw data were collected as PCAP (packet capture) files and exported as a CSV file using Wireshark. Next, the two attack models are discussed in the respective sub-sections.

#### 3.2.2. Protocol-Specific (RA) Attack Model Simulation

Two network models are designed for the protocol-specific attack simulation based on: (1) the number of nodes and (2) the state of nodes, each of them further divided into two use cases. The first use case of the first scenario comprises twenty nodes in the network, where one node is malicious and nineteen nodes are normal. In the second case of the first scenario, the network comprises fifty nodes, where two nodes are malicious and forty-eight are normal with one sink node in each case. The second scenario depends on the state of the node, which is either static or mobile. The first instance of the second scenario contains all nodes in a static disposition, while the second use case consists of a network with partially mobile nodes.

The rank attack is simulated from the protocol-specific attack category. The rank attack network model is cumulatively illustrated in Figure 2, where three nodes, 10, 19, and 20, broadcast the decreased rank and favorable characteristics through DIO messages to attract the network traffic. In the attack model, malicious nodes are placed near the root node strategically for the victim nodes to select them as parents. Algorithm 1 depicts the simulation of rank attack in the RPL-based IoT network in the Cooja simulator.
**Algorithm 1:** Protocol-specific RA Scenario
**1.**Input: rank attack building block, DIO control message**2.**Output: DIO message with the decreased rank **3.**    Begin**4.**   True: DIO with an illegitimately decreased rank**5.**       If**6.**        RPL_Conf_Min_HopRankInc = 0,**7.**        RPL_Max_RankInc = 0, **8.**        Infinite_Rank limited to 256, and**9.**        Rpl_recalculate_ranks = null, then**10.**        Child nodes select the parent,**11.**       Attack instigated**12.**     Else**13.**       False: node = benign **14.**    Until decreased rank attack is launched **15.**         Network = attacked**16.**End

#### 3.2.3. SN-Inherited (WHA) Attack Model Simulation

Two network models were designed for the SN-inherited attack simulation based on: (1) the number of nodes and (2) the state of nodes, each of them further divided into two use cases. The first use case of the first scenario comprises twenty nodes in the network, where two nodes are malicious and eighteen nodes are normal. In the second case of the first scenario, the network comprises fifty nodes, where two nodes are malicious and forty-eight are normal with one sink node in each case. Similar to the protocol-specific attack network model, the second scenario also depends on the state of the node, which is either static or mobile. The first use case consists of all static nodes, while the second use case consists of a network with partially mobile nodes.

The wormhole attack is simulated from the SN-inherited attack category. The attack model is presented in Figure 3, where two nodes, 16 and 26, form a tunnel between each other by probing using DIS messages and then sending and receiving DIO, DAO, and acknowledgment messages. In the attack model, malicious nodes are placed near the victim nodes to observe the attack effect. Consequently, the neighbor nodes 17–25 join the attacker node 16 by selecting it as a parent due to its illegitimate preferred parent characteristics, such as decreased rank value and shortest path to the root node. Similarly, nodes 27–30 also join the attacker node 26 for the same reasons mentioned earlier. Algorithm 2 demonstrates the simulation of SN-inherited wormhole attack in the RPL-based IoT network in the Cooja simulator.
**Algorithm 2:** SN-Inherited WHA Scenario
**1.**Input: wormhole attack building block**2.**Output: attacked RPL network **3.**    Begin**4.**   True: malicious nodes form tunnel via probing using DIS **5.**       If**6.**        Receive route requests,**7.**        Broadcast high-level capability, **8.**        Neighbor nodes overhear fake credentials,**9.**        Join the node as child nodes, **10.**        Drop the child node packets, then**11.**        Nodes = malicious **12.**     Else**13.**       False: node = benign **14.**    Until wormhole attack launched **15.**         Network = attacked**16.**End

### 3.3. Raw Data Collection

**Figure 4:**
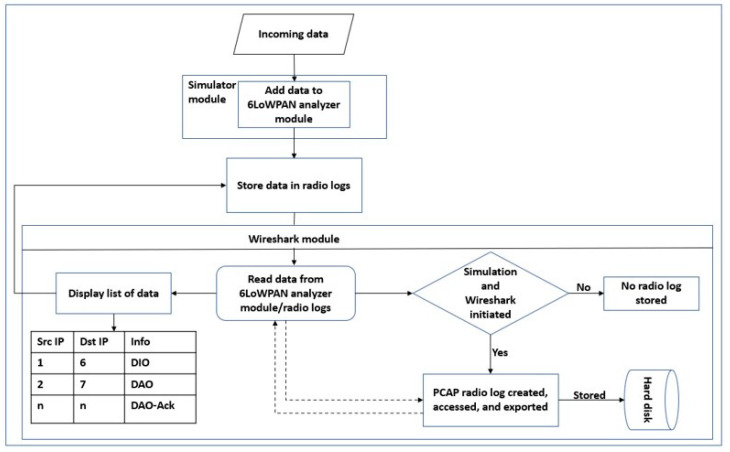
Workflow of data collection module.

After simulating our network models in different scenarios, the radio message tool was used to sniff and collect the radio messages transmitted between the nodes that would be analyzed with a 6LoWPAN analyzer with a PCAP feature incorporated into the tool. Figure 4 presents the workflow of the data collection module. Subsequently, the collected data were processed by the Wireshark software and saved in comma-separated values (CSV) format.

### 3.4. LIoTN-RPL Dataset Creation and Data Preparation

We used the Wireshark network analyzer to perform deep network traffic analysis and fragment the data to observe the traffic pattern for creating an extensive and all-inclusive dataset with appropriate feature vectors called the LIoTN-RPL dataset. As a result, the total extracted features counted to 210. We then performed the data cleaning process to delete duplicate features, resulting in the total number of features being reduced to 61, the majority of which had numeric values. Subsequently, the fixed features (with unchanging values) were removed from the dataset. Missing values were handled using the NaN as a replacement using the Pandas library in Python. Then, the categorical input features including source ID, destination ID, and protocol were encoded using one-hot encoding. One-hot encoding is a technique used in machine learning for converting categorical data into numerical data for ML-based models to successfully train them on the dataset. This is because ML models, particularly the ones involving classification algorithms for binary and multiclass problems, require the data to be in a uniform format for improving the model performance [30,31]. One-hot encoding achieves this by converting the categorical values into a numerical format. Therefore, we have used this approach to transform the categorical features in our dataset into numerical data for uniformity. Moreover, we have encoded the target labels as (0–2) for benign, rank, and wormhole attack traffic, respectively. The static node dataset contains 17,736 data instances and the mobile node dataset contains 13,326 data points.

#### Feature Engineering

Feature engineering techniques for machine learning are a fundamental element in machine learning but are usually neglected or conducted in an uninvolved manner. However, this step needs to be performed carefully because it plays a crucial role in the accuracy of the models Feature engineering involves various processes including feature selection, transformation, and normalization that lead to a prepared dataset for building the model. Given the nature of the dataset, the feature selection process was adopted in this paper. The broad categories of feature selection methods include supervised and unsupervised methods. As this paper proposes a supervised-learning-based model, the former methodology is preferred. Supervised-learning-based feature selection methods include wrapper, filter, embedded, and hybrid methods. In this paper, we have adopted one of the filter methods called the correlation matrix with heatmap for feature selection, and a set of best resulting features is presented in Table 3.

### 3.5. Multiclass Classification Model

In this paper, a multiclass classification model was proposed to address the rank and wormhole attacks in an RPL-based IoT network. The light gradient boosting machine model is leveraged to perform multiclass classification for classifying benign, rank, and wormhole target classes in the dataset. The model was developed for binary classification by Microsoft in 2016 as a lightweight variant of the gradient boosting method with underlying one-side sampling and exclusive feature bundling methods. The one-side sampling method called GOSS establishes and maintains the precise information gain by keeping the high gradient data instances a high priority and dropping the limited gradient data instances [32]. Equation (1) presents the mathematical form of the GOSS function. ${\hat{V}}_{j}$ presents the approximate variance gain over *A∪B* subset which is presented by *A_l_*, *A_r_*, *B_l_*, and *B_r_* in the equation while 1 − *a/b* indicates the normalization coefficient for the gradient sum. The ${\hat{V}}_{j}\left(d\right)$ is used to find the optimal split point for smart sampling of the dataset and for improving the model accuracy by focusing on the instances with large gradients. This also helps in reducing the complexity.

(1) V^jd=1n((∑xiϵAlgi+1−ab ∑xiϵBlgi)^2 /nljd+(∑xiϵArgi+1−ab ∑xiϵBrgi)^2/nrjd  

Secondly, the exclusive feature bundling method called EFB helps in minimizing the complexity by wrapping the exclusive features into a single feature. The histogram-based algorithms underlying the model help in improving the training time as well as they use less memory which is advantageous for LLNs such as IoT [33]. Therefore, this model is leveraged to perform multiclass classification followed by hyperparameter optimization and fine tuning.

## 4. Results and Discussion

### 4.1. Performance Evaluation Metrics

In this research study, we have considered several factors in determining the performance evaluation metrics to assess the performance of the proposed model. These metrics are based on the confusion metrics results that form a solid foundation for examining the classification-based ML models. Furthermore, we have also considered the type of classification, which is multiclass in this case, and which requires further parameters to counter any accuracy-related bias. Therefore, we have adopted cross-entropy, Cohn’s kappa, and Matthews correlation coefficient for extensive evaluation and validation. Finally, the performance of the model is compared with the benchmark research works and related classifiers.

Accuracy is the first evaluation metric used for the evaluation of the model’s performance. It calculates the number of correct predictions among all the predictions made by the model. Equation (2) presents the accuracy where *TP, TN, FP*, and *FN* denote true positive, true negative, false positive, and false negative, respectively.

(2) $$\frac{\left(TP+TN\right)}{\left(TP+TN+FP+FN\right)}\;$$

The second metric used for model performance evaluation is precision. It calculates the accuracy of each class using the parameters from the confusion matrix. Equation (3) presents the precision.

(3) $$\frac{\left(TP\right)}{\left(TP+FP\right)}\;$$

Recall, also known as the detection rate, is the ratio between the number of attacks detected by the system and the total number of attacks that are present in the dataset. Equation (4) calculates the detection rate.

(4) $$\frac{\left(TP\right)}{\left(TP+FN\right)}\;$$

Cross-entropy measures the extent to which the predicted probabilities match the given data and is used to quantify the cost of inaccurate predictions. The terms log loss and cross-entropy are used interchangeably; the lower the log loss, the better the model has performed. Cohn’s kappa addresses potential bias towards the major class (if any) by statistically measuring the vicinity of the predicted classes to the actual classes when compared with a random classification. Matthews correlation coefficient computes the correlation coefficient between the observed and predicted classifications within a range of +1, 0, and −1, where +1 indicates the ideal prediction model, 0 indicates random prediction, and −1 depicts inverted or reverse prediction. In this paper, we have used these metrics for the evaluation of the proposed model in addition to classic ML-based performance evaluators.

### 4.2. Results and Findings

For the proposed model to detect the RA and WHA in an RPL-based IoT network, we have performed substantial analysis using Python language. The model is trained on 70% of the training dataset and tested on the remaining 30% of unseen dataset. Furthermore, we have performed feature engineering and fine tuning to improve the accuracy of the model and compared it against our benchmark research as well as other ML classifiers to validate the obtained results.

**Figure 5:**
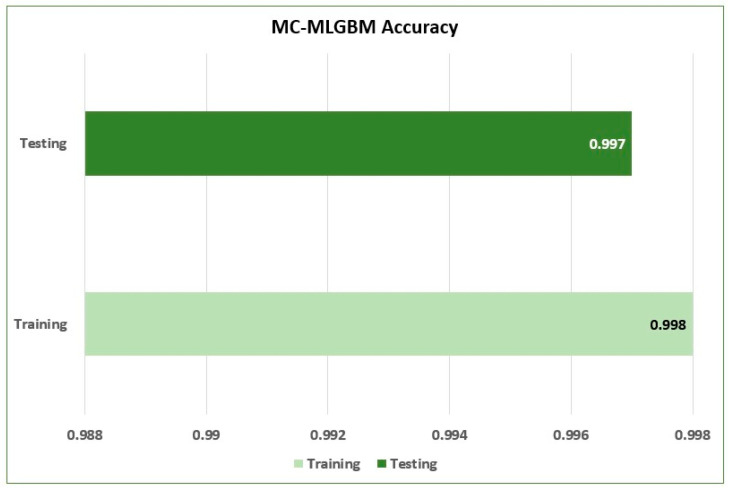
Training and testing accuracy of the proposed model.

The results obtained from the experiment exhibited promising results in both classic ML-based evaluation metrics and multiclass-related metrics. The model achieved a training accuracy of 0.998 and a testing accuracy of 0.997 for the detection of both types of attacks, as shown in Figure 5.

**Figure 6:**
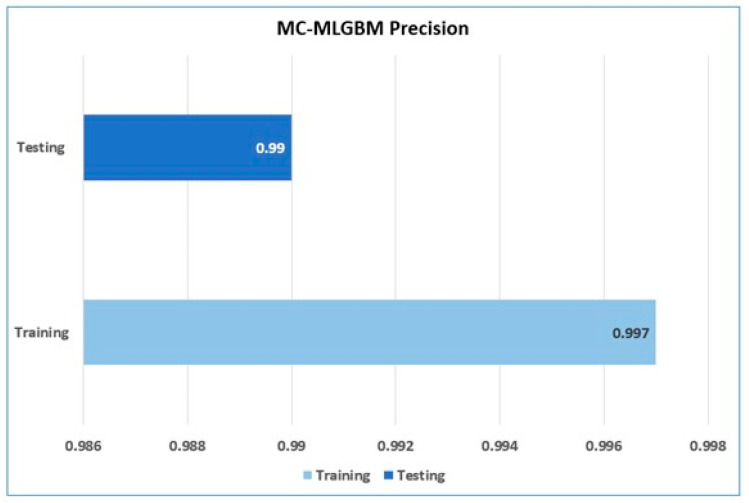
Training and testing precision of the proposed model.

The model achieved an average training precision of 0.997 and average testing precision of 0.99 as shown in Figure 6.

**Figure 7:**
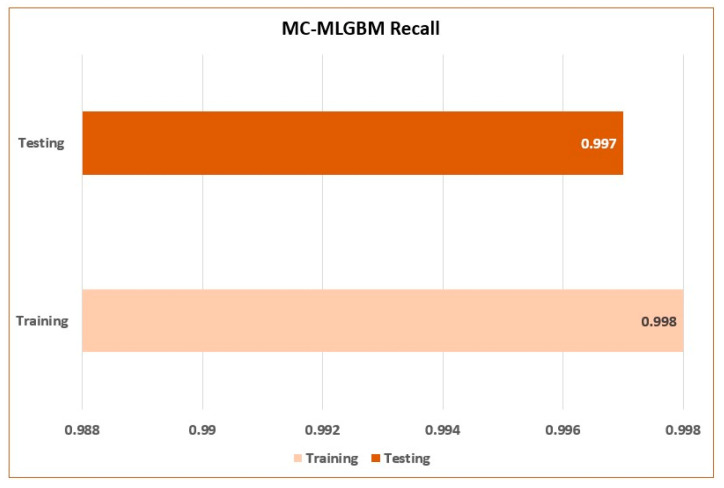
Training and testing recall of the proposed model.

The model achieved an average training recall of 0.997 and an average testing recall of 0.998 as shown in Figure 7.

Furthermore, the multiclass classification-related performance evaluation is summarized in Table 4. Table 5 presents the overall results and their comparison with other ML classifiers including ML-LGBM [13], gated recurrent unit-based DL (GRU-DL) [14], gradient boosting, XGBoost, and multiclass SVM, demonstrating that our proposed model MC-MLGBM outperforms the mentioned classifiers. Furthermore, the light gradient boosting model at the base of our multiclass classifier has a lower fitting time as compared to other classifiers, for instance, XGBoost, while it also distinguishes RA from WHA and separates the benign traffic traces from attack traffic traces in the LIoTN-RPL dataset. Mobility is also addressed in our study by extracting the mobility-induced dataset using the random waypoint mobility model [34,35] in the Cooja simulator.

The main findings from the results are summarized as follows:

- The results obtained from the experiments illustrate that the proposed model performs well in terms of addressing both the RPL-specific RA and SN-inherited WHA with respect to overall accuracy (99.7%), precision (99%), and detection rate (99.7%).
- The advanced metrics used for evaluating the multiclass classification show promising results where the model achieves low cross-entropy value (0.116), which indicates high accuracy. The high values of Cohn’s Kappa and MCC indicate that the model performs comparatively better.
- The above metrics also confirm the unbiased accuracy, which might have been present if only overall accuracy was used for evaluation.
- The proposed model outperforms benchmark research and classifiers in terms of accuracy, precision, and recall for two different types of attacks, that is, RPL-specific RA and SN-inherited WHA.
- The model achieves high performance during the learning phase, which is presented through the assessment of the model through the training set after fine tuning. The final evaluation conducted on the testing set shows enhanced performance in terms of attack detection through multiclass classification.

## 5. Conclusions

This paper proposed the machine-learning-based classification model called MC-MLGBM for the detection of RPL-specific RA and SN-inherited WHA in an RPL-based IoT network. For our study, we have generated the static and mobility-induced datasets gathered in a pool called LIoTN-RPL using the Cooja simulator for training and testing the models. This dataset was generated by simulating various network models depending on the number of nodes, and state of the nodes. The LIoTN-RPL dataset was used as a benchmark for our model to perform multiclass classification. Extensive analysis was performed to test the proposed model and compare with the benchmark research including binary light gradient boosting machines as well as other classifiers. The confusion matrix was used for analysis and the derived metrics such as accuracy, precision, and recall were used for model performance evaluation. The results were promising and better than our benchmarks in terms of multiclass classification of RA and WHA, achieving average accuracy, precision, and recall of 99.7%, 99%, and 99.7%, respectively, for the multiclass LIoTN-RPL dataset. We have further validated our model using metrics such as cross-entropy (0.116), Cohn’s kappa (0.93), and MCC (0.927) for multiclass classification and imbalanced class in the dataset to avoid biased accuracy results. The model outperforms overall when compared with benchmark research, binary classifiers, and multiclass SVM.

Although the proposed model shows promising results, there are some limitations to our work. Further developments are required in the LIoTN-RPL dataset. Currently, it contains benign traffic data, RA, and WHA attack data. More attacks from both RPL-specific and SN-inherited categories need to be simulated and collected in the LIoTN-RPL data pool for diversification, and further model evaluation. The future research direction is inspired by this limitation and the current promising results that are obtained for two attacks. In future, more attacks from both categories of RPL attacks will be considered by designing attack models, conducting simulation studies, dataset generation, and detection using the proposed model.

## Figures and Tables

**Table 1:** Summary of Related Works, Limitations, and Research Gaps.

Ref.	Dataset	Methodology	Evaluation Method(s)/Result	Limitation/Gap
[13]	Self-generated	An ML-based model for the detection of version number attacks in RPL-based IoT	Accuracy, precision, recall, F1 score, log loss	SN-inherited attacks were not considered, and mobility was not considered
[14]	Self-generated	A DL model using a gated recurrent unit network-based method to detect hello flooding attacks in the IoT network	Accuracy, mean squared error, mean absolute error, root mean square error	Protocol-specific attacks were not considered, mobility was not considered, DL-based methods require high computation and memory, scalability issues
[17]	Doesn’t apply/simulation study	Proposed to employ expected transmission count as a metric and developed trust-based technique for securing the routing topology	Packet delivery ratio, energy consumption, throughput, rank change	Mobility was not considered, uses security in the form of a chip with every node, and additional hardware required
[18]	Simulation study	A framework for securing vehicular ad hoc networks from data transmission attacks	False alarm probability, missing detection probability, velocity, end-to-end delay	Routing attacks were not considered, limited to vehicular ad hoc networks
[19]	Simulation study	Proposed a protocol to address jamming attacks and identify attacker nodes in vehicular ad hoc and IoT networks	Routing overhead, precision, recall, throughput	RPL-specific attacks were not considered
[22]	Simulation study	A security model based on dynamic and parametrized trust for IoT systems	Trust accuracy, trust value convergence, model resilience to change	Not suitable for routing attacks, RPL-specific and SN-inherited attacks were not considered, and mobility was not considered
[23]	Simulation study	Proposed two lightweight methods based on elimination and shielding strategies to address version number attacks in RPL networks	Power consumption, control message overhead, packet delivery ratio	SN-inherited attacks were not considered, and mobility was not considered
[15]	Self-generated	An intrusion detection-based technique using neural networks to address routing attacks in RPL-based wireless sensor networks	Not mentioned	Mobility was not considered; placement strategy was not discussed; evaluation metrics were not mentioned
[24]	Self-generated	Proposed to address RPL-specific attack called version number attack using a beacon, routing metric, and ML classification-based framework	Accuracy, precision, recall, specificity	SN-inherited attacks were not considered, and mobility was not addressed

**Table 2:** The datasets from different network models and scenarios.

Network Models for Simulation	Scenarios		Dataset	Total
	No. of Nodes	Node State		
**Benign network model**	1st case: 20 nodes; 1 sink node, 19 sender nodes, all benign 2nd case: 50 nodes; 1 sink node, 49 sender nodes, all benign	1st case: static 2nd case: mobile	1st case: 6250 2nd case: 7512	13,762
**Protocol-specific attack network model (rank attack)**	1st case: 20 nodes; 1 attacker node, 19 benign nodes with 1 sink node 2nd case: 50 nodes; 2 attacker nodes, 48 benign nodes with 1 sink node	1st case: static 2nd case: mobile	1st case: 7732 2nd case: 3457	11,189
**SN-inherited attack network model (wormhole attack)**	1st case: 20 nodes; 2 attacker nodes, 18 benign nodes with 1 sink node 2nd case: 50 nodes; 2 attacker nodes, 48 benign nodes with 1 sink node	1st case: static 2nd case: mobile	1st case: 3754 2nd case: 2357	6111 Total static: Total static: 13,326

**Table 3:** The selected features for model development.

No.	Selected Features	Feature Name
**1**	src.6lowpan	Source ID
**2**	dst.6lowpan	Destination ID
**3**	dio.rank	DIO rank
**4**	dao.ack	DAO acknowledgment
**5**	ack	Acknowledgment
**6**	udp	UDP
**7**	maxrankinc	Maximum Rank Increase
**8**	minhoprankinc	Minimum Hop Rank Increase
**9**	rerr	Rank error
**10**	diointervalmin	Minimum DIO interval
**11**	dioredconst	DIO redundancy constant
**12**	protocol	ICMPv6, UDP, and IEEE 802.15.4 protocols
**13**	rank	Rank value
**14**	lost	Lost packets
**15**	hopcount	Hop count
**16**	ipv6hoplim	Hop limit
**17**	wpanseq.no	6LoWPAN sequence number
**18**	dio.dst	DIO destination
**19**	mesgs	Message type
**20**	dis	DIS
**21**	diointervalmin	Minimum DIO interval

**Table 4:** Performance Analysis of the Proposed Model.

Evaluation Metric	Train/Test	Result
**Accuracy**	Training	0.998
	Testing	0.997
**Precision**	Training	0.997
	Testing	0.99
**Recall**	Training	0.998
	Testing	0.997
**Cross entropy**		0.116
**Cohn’s Kappa**		0.93
**MCC**		0.927

**Table 5:** Comparison of the Proposed Model with Benchmark and Other Classifiers.

Model	Attack		Accuracy	Precision	Recall	Cross Entropy	Cohn’s Kappa	MCC
	Protocol-Specific	SN-Inherited						
Proposed MC-MLGBM	✓	✓	0.998	0.997	0.998	0.116	0.93	0.927
ML-LGBM	✓	✗	0.981	0.97	0.96	0.1289 log loss	-	-
GRU-DL	✗	✓	0.99 for selective features	-	-	-	-	-
MC-SVM	✓	✓	0.965	0.95	0.962	0.159	0.89	0.90
GB	✓	✗	0.99	0.98	0.98	-	-	-
XGBoost	✓	✗	0.988	0.977	0.983	-	-	-

## Data Availability

Data will be available on request.
